# Nanoporous Polymers Based on Liquid Crystals

**DOI:** 10.3390/ma11010104

**Published:** 2018-01-11

**Authors:** Jody Lugger, Dirk Jan Mulder, Rint Sijbesma, Albert Schenning

**Affiliations:** 1 Laboratory of Supramolecular Polymer Chemistry, Department of Chemical Engineering and Chemistry, Eindhoven University of Technology, P.O. Box 513, 5600 MB Eindhoven, The Netherlands; j.a.m.lugger@tue.nl; 2 Institute for Complex Molecular Systems, Eindhoven University of Technology, P.O. Box 513, 5600 MB Eindhoven, The Netherlands; 3 Laboratory of Stimuli-Responsive Functional Materials and Devices, Chemical Engineering and Chemistry, Eindhoven University of Technology, P.O. Box 513, 5600 MB Eindhoven, The Netherlands; D.J.Mulder@tue.nl; 4 Dutch Polymer Institute, P.O. Box 902, 5600 AZ Eindhoven, The Netherlands

**Keywords:** liquid crystals, nanoporous membranes, filtration, separation, adsorption, ion conductivity, polymer network

## Abstract

In the present review, we discuss recent advances in the field of nanoporous networks based on polymerisable liquid crystals. The field has matured in the last decade, yielding polymers having 1D, 2D, and 3D channels with pore sizes on the nanometer scale. Next to the current progress, some of the future challenges are presented, with the integration of nanoporous membranes in functional devices considered as the biggest challenge.

## 1. Introduction

The need for more efficient and cheaper ways to remove pollutants from water is becoming increasingly urgent, as clean water is turning into a scarcity rather than a commodity [1]. Some of the biggest problems are the build-up of contaminants in current water resources, and the depletion of groundwater [2,3,4]. To meet the increasing demand for fresh water, membrane filtration is often employed for pre-treatment as well as for the final step in producing fresh water [5]. At the moment the membrane separation field is dominated by nano-filtration (NF) membranes, with 1–5 nm heterogeneous pores, and ~1% porosity [6], and by reverse osmosis (RO) membranes, which are composed of even smaller pores. RO membranes outperform NF membranes, with a typical rejection of >99% of all matter, except for soft monovalent ions. Typically, pressures up to 100 bar are applied, making this process costly in terms of energy consumption.

**Figure 1:**
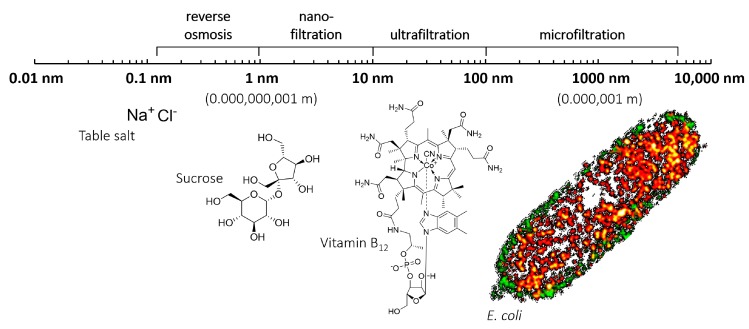
Porosity range of different types of filtration. Reverse osmosis removes virtually all matter, while for some fresh water sources microfiltration is sufficient to produce drinking water by simply removing bacteria. Harmful small molecules and toxins need to be removed by for example ultra- or nanofiltration. For *E. coli* a single cell super-resolution fluorescence image is shown [8], licensed under a Creative Commons Attribution 3.0 Unported License.

The clear advantage of nanoporous polymer networks compared to standard mesoporous materials is their better selectivity because of reduction of pore size, see Figure 1. NF and RO membranes typically have 10^16^ effective pores per 1 m^2^ [7]. However, there is still considerable room for improvement because these pores constitute less than 0.1% of the membrane surface. Moreover, performance could be further improved with more uniform pore sizes. Wider, but more uniform pores will result in a higher flux of filtrate, increased kinetics, and better selectivity.

Lithographic top-down methods are currently being developed that are able to produce features on the nanometer length scale [9]. On the other hand, with inorganic nanoporous materials such as metal-organic frameworks (MOFs) and zeolites already small monodisperse sub-1-nm pores can be accessed, and these robust materials have extensively been used for their nanoporosity [10,11,12,13].

Using self-assembly is a third strategy to fabricate nanoporous films, with the benefit that such a bottom-up approach does not require additional machinery. Furthermore, the pore sizes that can be achieved with self-assembly are in principle only limited by the size of the building blocks, molecules or polymers, and the materials can directly be processed into thin films. For example, utilising the self-assembly properties of block copolymers, porous films were prepared with pore sizes down to the nanometer scale by selective removal of one of the incompatible blocks [14]. However, the smallest attainable pore size using block copolymers is limited to the shortest block length that still gives microphase separation, which is estimated to approach 2 nm feature sizes [15]. However, by using an A-B block copolymer, combined with the covalent attachment of a polyelectrolyte brush on the pore lining, polymer membranes have been fabricated with sub-nanometer size selectivity [16].

Another elegant and appealing bottom-up approach for fabricating nanoporous membranes is by using self-organising polymerisable liquid crystals (LCs). In the liquid crystal phase, molecules combine the mobility of isotropic liquids with a degree of order that may approach that of solids. LCs exhibit orientational order and can be organised in well-ordered morphologies over large areas, with features in the nm-range. Additionally, LCs have the ability to align in monodomains, and they can readily be polymerised to fixate their morphology. Upon selective removal of specific parts of such polymerised LCs, they can yield nanoporous films with mono-disperse pores distributed in an ordered fashion. In this manner, membranes with a high density of sub-1-nm pores can easily be produced. Hence, polymerisable LCs are excellent building blocks to prepare nanoporous polymers.

Different types of liquid crystal phases have been identified, among which lyotropic phases (with solvent-induced liquid crystallinity), and thermotropic phases (with thermally induced liquid crystallinity) are the best known. Liquid crystals have been used to prepare polymer films with a wide range of applications in, e.g., sensors [17], optical elements [18,19], and actuators [20,21,22]. While small molecules provide mobility to LCs, they do not provide the mechanical properties required for membrane applications. Therefore, a curing step is required to fixate the morphology of the LC phase, before their anisotropic properties can be used for the fabrication of, e.g., nanoporous polymer films or coatings. Fixation can be attained by providing the molecules forming the LCs with reactive end groups. Because of its speed and robustness, photo-initiated free radical polymerisation of acrylates/methacrylates is probably the most frequently applied method for fixating LC morphologies [23,24]. However, there are many alternative fixation methods available to obtain LC networks, including acyclic diene metathesis (ADMET) [25,26,27], thiol-ene chemistry [28], cationic polymerization of epoxides [29], coumarin dimerization [30], free radical polymerization of dienes [31], and hydrosilylation of alkenes [32,33]. Upon polymerisation, highly cross-linked networks can be obtained. Although the anisotropic polymer networks retain the order of the LC phase, they often lose their mobility; yet the label liquid crystal networks (LCNs) is used.

The initial strategy to obtain nanoporous membranes from LCs was to produce porous LC networks directly from suitable monomers. For example, the early work from the group of Beginn relied on the ion-conductive properties of crown ethers to induce porosity [34,35]. Later on, however, the paradigm changed, and in more recent work pores are usually introduced in the membranes after fixation of the LC morphology. Nanopores can be introduced in the LC network in many ways, which can be classified into two distinct approaches: (1) disrupting non-covalent bonds such as hydrogen bonds inherent in the network, creating voids because of for example Coulomb repulsion of the resulting charged head groups [36,37]; and (2) removal of a covalently, or non-covalently, linked template from the network, creating uniform pores [24,26,27,38,39,40,41,42].

In recent years, much progress has been made in the development of nanostructured films based on polymerisable thermotropic liquid crystals, and for the formation of nanoporous polymer films discotic LCs have been investigated in particular. Improvements in these methodologies gave rise to anisotropic porous polymers with pore sizes down to ~1 nm [23,26,28,43]. For the fabrication of nanoporous polymers, the use of both lyotropic liquid crystals and thermotropic liquid crystals has been reported. When lyotropic LCs are used, the size of the pores in the polymerised network is determined by the amount of water (solvent) present in the organised mesophase; this approach has been reviewed by Wang et al. [44].

**Figure 2:**
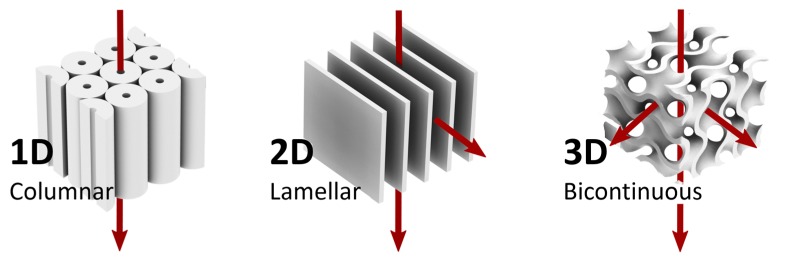
Nanoporous liquid crystal networks having various morphologies. Columnar and hexagonal yield 1D pores, lamellar or smectic mesophases yield 2D pores, and bicontinuous mesophases give a 3D pore geometry.

The morphology of the nanopores is determined by the type of mesophase, Figure 2. One dimensional (1D) pores can be obtained from hexagonal or columnar mesophases in lyotropic LCs or thermotropic discotic LCs [45,46]. In contrast to this, lamellar (2D) morphologies can be obtained using thermotropic calamitic LCs polymerised in the smectic mesophase, while polymer networks containing tortuous three dimensional (3D) pores have been prepared by polymerising lyotropic liquid crystals in the bicontinuous cubic mesophase.

In the sections below, recent developments on 1D, 2D, 3D nanoporous polymers will be reported, and items such as LC alignment, pore surface chemistry, and applications, related to each type of morphology (Figure 2) will be discussed.

## 2. Nanoporous LC Networks

### 2.1. One-Dimensional Pores

**Figure 3:**
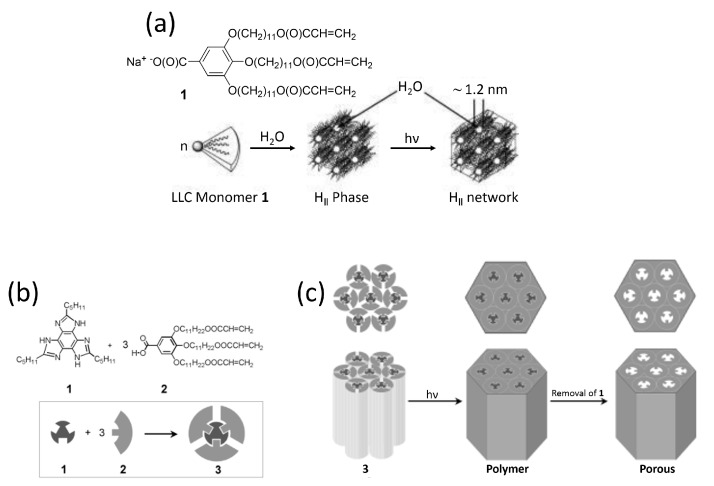
The fabrication of nanoporous hexagonal liquid crystal networks. (**a**) Chemical structure of the lyotropic gallate moiety and stepwise formation of a porous LC network; (**b**) The chemical structures of the template and gallic acid derivative; (**c**) Procedure for the fabrication nanoporous columnar polymer, including the self-assembly, polymerisation, and removal of the template moiety. Figures were partly reproduced from [23,49], licensed by John Wiley and Sons, Inc. (Hoboken, NJ, USA).

Materials with 1D, cylindrical pores can be prepared from disk-shaped (discotic) mesogens which typically have a planar aromatic core surrounded by flexible alkyl tails. In this type of molecules, attractive interactions between aromatic cores govern the self-assembly [47,48]. Preferential stacking of the disks provides the driving force to give columns with a high persistence length, while the peripheral tails prevent crystallisation. The combination of the two effects affords the discotic mesophase. The shape of the discotic mesogen and its possible tilt in the column will determine the type of unit cell of the columnar phase; hexagonal, rectangular, and square columnar phases have been described [48]. Two different approaches, each using a wedge-shaped gallic acid derived monomer, have widely been followed in the production of nanoporous materials with cylindrical pores. The first approach is based on deprotonation of the acid yielding a gallate salt, Figure 3a. The salt can be used either as a thermotropic [43], or lyotropic liquid crystal [49,50]. Polymerization of the lyotropic mesogen in an inverted hexagonal phase results in cylindrical aqueous channels. When polymerised as a thin film on a porous support, such a material can potentially be used as a selective layer for nanofiltration [49]. The second approach utilises a template molecule which is hydrogen bonded to three gallic acid monomers to form an AB_3_ complex, Figure 3b, yielding a nanoporous material after fixation of the morphology by polymerisation followed by selective removal of the template molecule (porogen).

**Figure 4:**
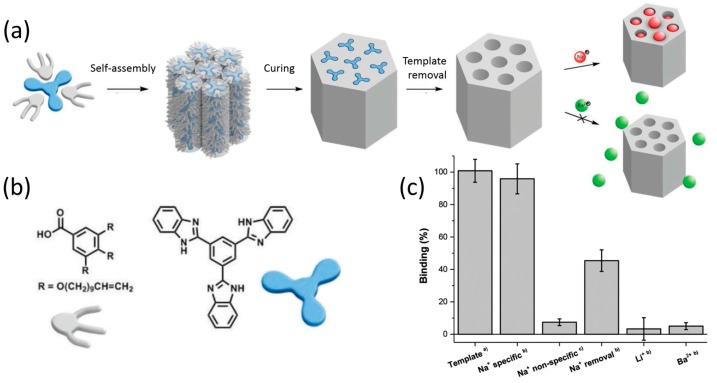
Nanoporous polymers using a gallic acid derived monomer and tris-benzimidazole template. (**a**) Scheme illustrating monomer self-assembly, polymerisation, template removal, and selective ion uptake; (**b**) Monomer and template used for the self-assembly; (**c**) Size-selective adsorption of ions as measured by using a quartz crystal microbalance. Adapted from [26], under the terms of a Creative Commons Attribution Non-Commercial License CC BY-NC.

Smaller molecules (2-amino alcohols) have also been used as templates in a 1:1 stoichiometry [42,51,52,53]. The order in the material after template removal was improved by using diene instead of acrylate reactive groups [53]. Membranes with aligned helical pores were obtained when a 2-amino alcohol template was used to translate its chirality to the polymer host, and the LC phase was aligned in a magnetic field [42]. Using a similar template-approach, Bögels et al. prepared a nanoporous polymer based on hydrogen-bonded tris-benzimidazole—gallic acid mesogens (Figure 4a) [26]. The reactive gallic acid derivatives were cross-linked with an acyclic diene metathesis reaction, and after removal of the benzimidazole porogen, a nanoporous material was obtained. The small anionic nanopores selectively take up small monovalent metal ions over bigger monovalent or multi-valent cations (Figure 4b).

Using unsaturated fatty acids together with the previously mentioned benzimidazole template, Feng et al. prepared nanoporous polymers with pores 1.2–1.5 nm in diameter [54]. The hydrogen-bonded fatty acid/template complex formed a hexagonal mesophase that was copolymerized with additional bifunctional monomers to obtain a crosslinked network. The benzimidazole template was washed out under alkaline conditions in DMSO to obtain anionic nanopores in which a variety of dyes was absorbed with size and charge selectivity; only cationic dyes less than 1.8 nm in size were adsorbed. The pores in the material were retained as long the material was kept in an aqueous environment. Complete drying of the material led to the irreversible collapse of the pores.

**Figure 5:**
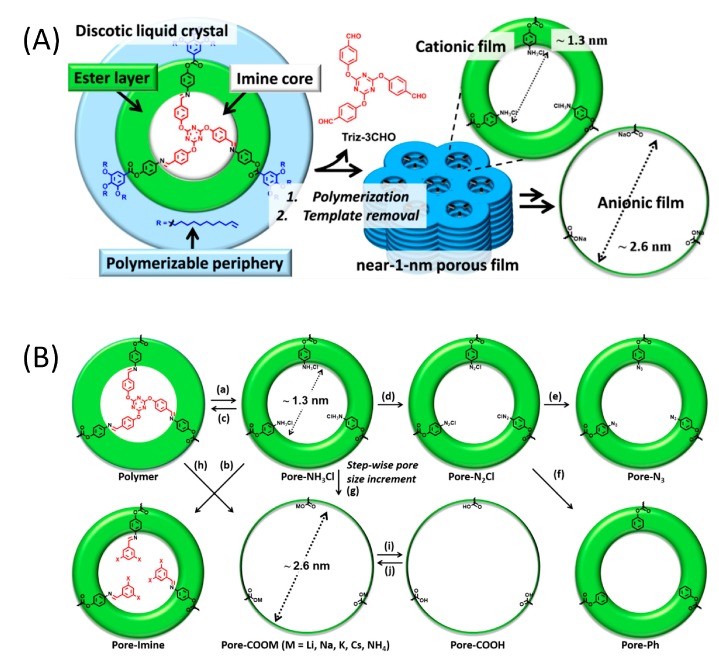
Chemical pore modification of a columnar hexagonal liquid crystal polymer. (**A**) Stepwise fabrication of a nanoporous polymer with smaller cationic, or bigger anionic pores; (**B**) Scheme of pore chemistry, starting from non-porous polymer. (a) Hydrolysis of the imine linkages of the native polymer yielding the nanoporous polymer. (b) Reacting the amino groups in the pores with different aldehydes. (c) Reincorporating the template aldehyde yielding the original polymer. (d) Conversion of the ammonium groups in the pores to the corresponding diazonium salt. (e,f) Reacting the diazonium salt with NaN_3_ and H_3_PO_2_ to fabricate porous polymers with neutral azide (–N_3_) and phenyl (–Ph) groups at the pore surface. (g) Hydrolysis of the ester groups present in the inner core of the polymer to furnish a porous polymer containing bigger anionic pores. (h) Hydrolysis of the native polymer to directly form the porous polymer with big anionic pores. (i) Acidic treatment of the anionic pore interior to yield neutral –COOH groups in the pores. (j) Treatment of the pores lined with –COOH groups with hydroxide salts of Li^+^, Na^+^, K^+^, Cs^+^, and NH_4_^+^ yielding anionic pores with different counterions. Adapted from [27], under the terms of a Creative Commons Non-Commercial No Derivative Works (CC-BY-NC-ND) Attribution License.

Recent developments illustrate the versatility of 1D nanoporous polymer materials. Films using new pre-mesogens and templates [28], a nanoporous superlattice [55], and adjustable chemistry have been prepared [27], and responsive pores have been developed by using metallomesogens and azobenzene in the fabrication of light responsive nano-gates or nano-valves [56]. Recently, Gracia et al. showed that columnar 1D porous polymers can serve as scaffolds for preparing nanometer-sized silver nanoparticles [28]. After inducing porosity, the pores were filled with a silver salt followed by a reduction step to yield the final particles distributed in the film. Bhattacharjee et al. reported the fabrication of a nanoporous Col_hex_ LC network that could selectively be converted into a porous polymer with small cationic pores, or larger anionic pores, Figure 5A [27]. Furthermore, they showed that the pore interior could be chemically modified using different chemical reactions, Figure 5B, e.g., an azo functionalized pore surface was obtained. The ability to customise the pore surface significantly increases the scope of nanoporous materials.

A major challenge to the application of columnar hexagonal mesophases for nanoporous membranes is to control alignment of the phase. The columns in the film must be oriented orthogonal to the surface (homeotropically) prior to polymerisation in order to obtain pores that run from top to bottom of the membrane. Planar alignment of the columns would lead to an increased transport resistance of the material. Various approaches have been used to tackle this challenge. Yoshio et al. prepared ion conducting membranes with specific alignment of columns by altering the surface chemistry of the substrate [40]. The importance of alignment was demonstrated by the ion conductivity, which was three orders of magnitude higher in the homeotropically aligned membranes than in membranes with planar alignment of columns.

**Figure 6:**
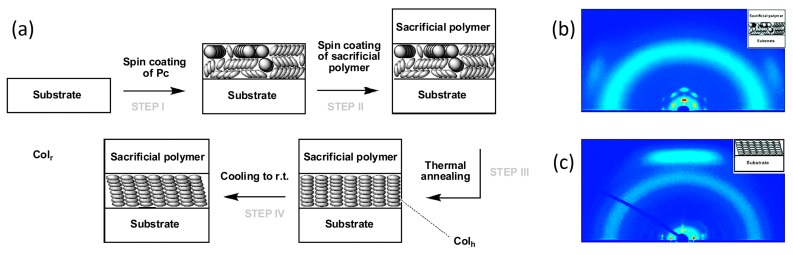
The alignment of hexagonally ordered liquid crystals by coating with a sacrificial polymer. (**a**) Schematic of the steps taken to align a Col_hex_ LC film on a solid substrate; (**b**,**c**) 2D grazing incidence X-ray diffractograms of LC film before (**b**) and after (**c**) the homeotropic annealing step. Reprinted (adapted) with permission from [61]. Copyright 2009 American Chemical Society.

Technically low demanding methods are known to align and reorient LCs, e.g., by using surface confinement [57,58], or by photo-aligning the LC [59,60]. Probably the method with the best potential for application consists of matching the interfacial energy with the liquid crystal [37,61]. This approach is highlighted by the work of Pouzet et al., who used a sacrificial polymer top layer as a convenient way for obtaining homeotropically aligned discotic LC phases [61]. Spin-coating an alkoxy phthalocyanine on a Si/SiO_2_ substrate gave planar alignment with small, randomly oriented domains. However, by applying a polyvinyl phenol top layer, the LC reorients to a homogeneous homeotropic alignment after thermally annealing the sample, Figure 6a. X-ray diffractograms before and after the procedure, Figure 6b,c, verified that the initial planar alignment (two diffraction spots on the side in the wide-angle range) reoriented into a homeotropic state (intense diffraction spot on top).

**Figure 7:**
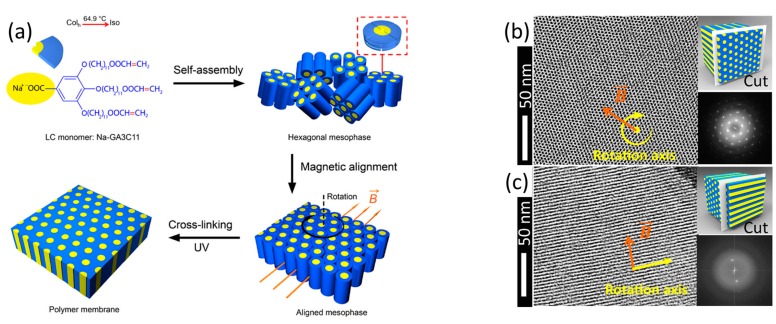
The alignment of hexagonal ordered liquid crystals in a magnetic field. (**a**) Schematic representation of the alignment procedure; (**b**,**c**) Transmission electron microscopy images of the aligned material microtomed along (**b**) and perpendicular (**c**) to the columns. Adapted from [43], under ACS Author Choice License.

Alignment in magnetic fields has emerged as another powerful method to obtain homeotropic films of discotic LCs and has culminated in the fabrication of perfectly organised nanoporous material based on the previously discussed sodium gallate salt, see Figure 3a and Figure 7, [42,43,54,62,63,64]. Highly aligned monodomains were obtained by rotating the film inside a 0–6 T magnetic field, prior to curing the morphology by the free radical polymerisation of pendent acrylates, as illustrated by transmission electron microscopy, Figure 7b,c.

The alignment of LC phases with high order, discotic LCs in particular, has proven to be challenging. However, with the wide range of alignment methods now being reported it is expected that it is a matter of time before these highly ordered materials will find applications.

### 2.2. Two-Dimensional Pores

Two-dimensional pores obtained from lamellar or smectic mesophases display extremely high tortuosity. Smectic liquid crystals have the benefit that they can be aligned using well-known alignment techniques [65], typically used in the liquid crystal display industry. Traditionally, ion conducting membranes have been prepared from either low molecular weight smectic liquid crystals [66,67,68,69,70], or from polymers [33,71]. Although these materials show unique anisotropic ionic conducting properties, they have a low degree of porosity and mechanical strength. With the use of polymerisable hydrogen-bonded reactive mesogens [72], the LC morphology was fixated in the resultant polymer network by rapid photopolymerisation of the LC monomers. After polymerisation of the mesogens in the desired mesophase, the hydrogen bonds can be disrupted inducing nanoporosity, yielding a variety of porous polymer networks.

**Figure 8:**
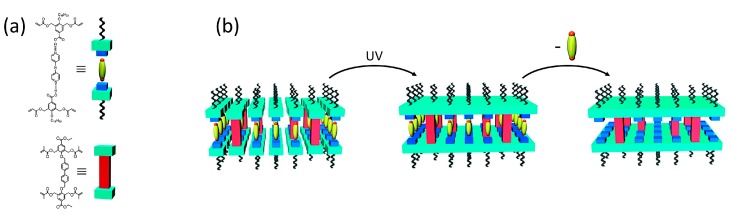
Smectic LC based nanoporous membrane. (**a**) Structures of the used supramolecular monomers; (**b**) Fixation of LC morphology via a UV curing step followed by the formation of the nanopores by removal of the dipyridyl template, a tetra-methacrylate ‘nanopillar’ was used to provide structure and layer integrity. Reprinted (adapted) with permission from [73]. Copyright 2008 American Chemical Society.

Without a linking unit between the lamellae of a polymerised smectic material, separation of the layers can occur when the hydrogen bonds are broken, leading to the disintegration of the material. In 2008, Kishikawa et al. incorporated reactive ‘nanopillars’ to hold the layers of a smectic polymer film together after removal of a hydrogen-bonded template (porogen), Figure 8a [73]. A mixture containing the hydrogen bonded smectic LC monomer and the covalent nanopillar monomers was polymerised in the smectic mesophase after which the template was removed by washing with a hydrochloric acid solution, Figure 8b, with maintained order as was shown with X-ray diffraction.

In a similar approach, Gonzalez et al. presented an anisotropic expansion of smectic films from dimerised benzoic acid derived LC monomers [36]. In this work, nanopores of approximately 1 nm were formed by breaking the hydrogen bonds with aqueous base. During this treatment, the material expanded approximately 50% perpendicular to the molecular director, while along the director nearly no change occurred [74]. After alkaline treatment of the hydrogen-bonded network, a nanoporous polymer containing two-dimensional nanopores with an anionic charge was obtained, which can be used as an efficient adsorbent for cationic species [75]. Small cationic dyes like methylene blue were adsorbed with a high occupation level (980 mg of dye per gram of material), whereas anionic methyl orange was not adsorbed. Large cationic dendrimers were adsorbed with similar efficiency as methylene blue, demonstrating charge selectivity.

**Figure 9:**
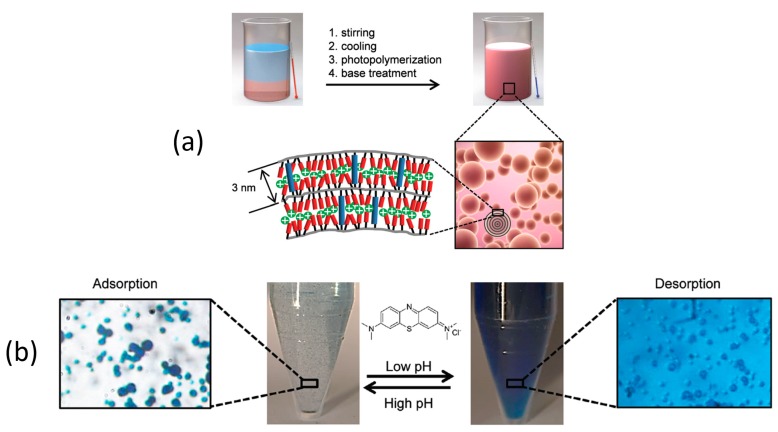
Nanoporous particles as adsorbents. (**a**) Illustration of polymer particle preparation from a polymerisable smectic liquid crystal; (**b**) Adsorption/desorption of methylene blue by the nanoporous particles. Adapted from [76]. Copyright 2016 The Royal Society of Chemistry (RSC).

The smectic nanoporous polymer films discussed above were produced in liquid crystal cells. This method limits the scalability of the production. Therefore, a suspension photopolymerization was developed to prepare small adsorptive particles from a similar LC monomer mixture, Figure 9a [76]. Using this polymerisation technique, the production of the adsorbent could be scaled up. The particles obtained with this method were a few micrometres large and had a radial alignment of the molecular director. Consequently, the nanopores were oriented concentrically. Swelling of the particles upon alkaline treatment allowed the formation of the pores, and a maximum of 887 mg methylene blue could be adsorbed per gram of particles. The reduction in capacity compared to the films (980 mg/g) was attributed to the concentric alignment of the pores affecting the adsorption equilibrium. Despite the unfavourable alignment, the particles show faster adsorption kinetics than the films, and the dye was released efficiently at low pH, Figure 9b.

**Figure 10:**
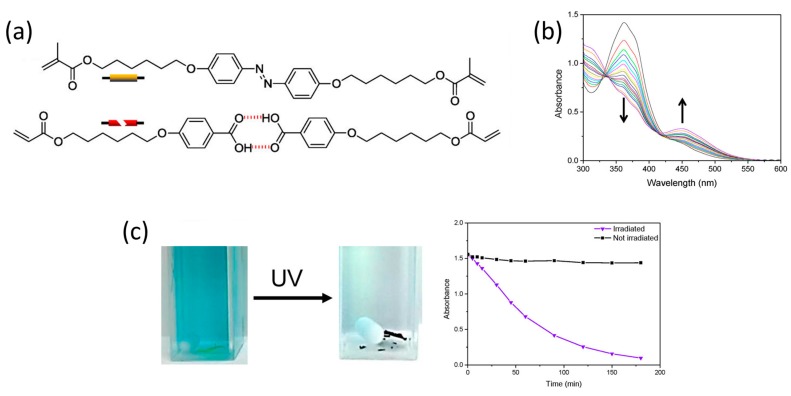
A photo-actuated porous adsorbent. (**a**) The chemical structures of the monomers, a trans–cis isomerizable crosslinker was used; (**b**) Monitoring azobenzene isomerization with UV/Vis; (**c**) Cuvettes containing a methylene blue solution at pH 9.5 and the photoresponsive adsorbent before and after UV exposure. The graph shows the adsorption over time for both cuvettes. Reprinted (adapted) with permission from [77]. Copyright 2015 American Chemical Society.

By substituting the crosslinker by an azobenzene variant, Figure 10a, a photoresponsive nanoporous LC network was obtained [77]. Azobenzenes can undergo trans-to-cis isomerisation when exposed to UV-light, Figure 10b. The isomerisation led to a small change in the lamellar spacing of the nanoporous smectic network and a change in pK_a_ of the benzoic acid moieties caused by the change in dipole moment when azobenzene isomerises to its cis-form. In this way, the hydrogen bonds could be selectively broken under the influence of light at pH 9.5. Under identical conditions, methylene blue was adsorbed in situ, Figure 10c.

Lamellar nanoporous polymers have also been used as a nanocomposite hybrid material, where the porous 2D layers act as a confined scaffold in which metal nanoparticles can be grown [78,79].

**Figure 11:**
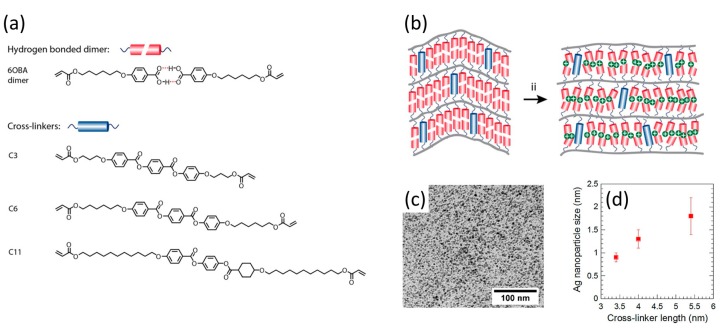
Synthesis of Ag nanoparticles inside 2D lamellae of a smectic LC network. (**a**) The molecular structure of the pore interior; (**b**) Schematic of converting the pore interior to the metal salt; (**c**) TEM image of particle distribution in the LC scaffold; (**d**) Mean particle size as a function of cross-linker length. Adapted from [80], under the terms of a Creative Commons Attribution-NonCommercial 4.0 International (CC BY-NC 4.0) License.

The same hydrogen bonded reactive liquid crystal with a variety of crosslinkers of different lengths, Figure 11a,b, has been used by Dasgupta et al., to make nano-sized pores that were used for the synthesis of silver nanoparticles [80,81]. By varying the length of crosslinker, the size of the particles was controlled, Figure 11d. The method involves the ion exchange of potassium ions with silver ions after formation of the pores, Figure 11b. Subsequently, the silver ions were reduced using sodium borohydride. The size of the particles was evaluated by various microscopy techniques and X-ray scattering and spectroscopy techniques, Figure 11c. Precise control over the size of nanoparticles is desired to tailor the optical and catalytic properties.

The lamellar anisotropy of 2D LC networks can be used to provide highly ion-conductive patches separated by less mobile low-conductive lamellae. For example, Liang et al. studied the anisotropic anhydrous proton conduction in a smectic liquid crystal and showed that increasing the crosslink density results in more ordered 2D nanostructures with a concomitant increase in proton conductivity [82]. Furthermore, the polymer films showed anisotropic proton conductivity with 54 times higher conductivity in the direction perpendicular to the molecular director.

**Figure 12:**
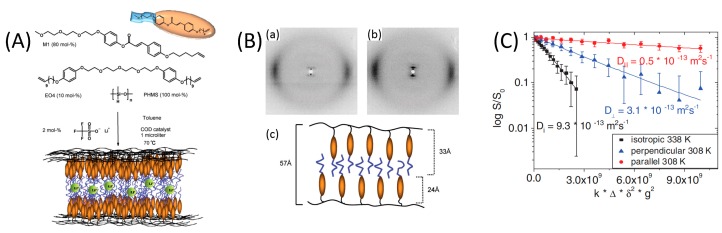
Synthesis of an elastomer loaded with Li-ions from a smectic LC network. (**A**) The used LC monomers and Li-salt, and an illustration of the conductive LC elastomer; (**B**) XRD pattern of elastomer without Li (a) and with Li incorporated (b) including a schematic of the interdigitated SmA layers (c); (**C**) Measurements of ^7^Li diffusion coefficients parallel and perpendicular to the director of the Li-elastomer at 308 K, and in the isotropic phase at 338 K. Adapted from [33], licensed by John Wiley and Sons, Inc. (Hoboken, NJ, USA).

Ramón-Gimenez et al. presented an LC elastomer with a smectic A phase loaded with a lithium salt prior to fixation, Figure 12 [33]. X-ray analysis confirmed that the LC phase and macroscopic orientation were retained in the network containing Li-salt, Figure 12B. By ^7^Li-NMR the lithium diffusion coefficients were determined parallel to the macroscopic orientation of the lamellae and perpendicular to the orientation, which revealed diffusional anisotropy in line with the material’s anisotropy. Lithium diffusion along the lamellae was found to be 6 times faster compared to perpendicular to the lamellae, Figure 12C. The lithium diffusion coefficient for the isotropic phase was found to be three times higher than the optimal anisotropic diffusion coefficient. Their demonstration of an anisotropic lithium ion conducting LC elastomer is promising for the development of LC networks as Li-ion electrolytes.

Overall, the formation of 2D porous LC networks is well established. And in the last years, this methodology has advanced from a conceptual novelty to an applicable tool in the materials science.

### 2.3. Three-Dimensional Pores

Despite the high degree of order currently achievable using hexagonal columnar phases, the difficulty of obtaining large-scale alignment (>micrometre) has limited their applicability for a long time. However, in applications that do not require the film to be anisotropic, bicontinuous mesophases can be used as a basis, and the requirement for LC alignment is circumvented. Bicontinuous mesophases are easy to process and provide continuous channels across the membrane without the need for alignment. A number of different monomeric lyotropic liquid crystals have been used for the preparation of nanoporous polymer networks with a 3D geometry [83,84,85], and bicontinuous LC films have already successfully been applied as an active layer for a size discrimination filter, which uses the continuous small-diameter channels of the LC network [86].

**Figure 13:**
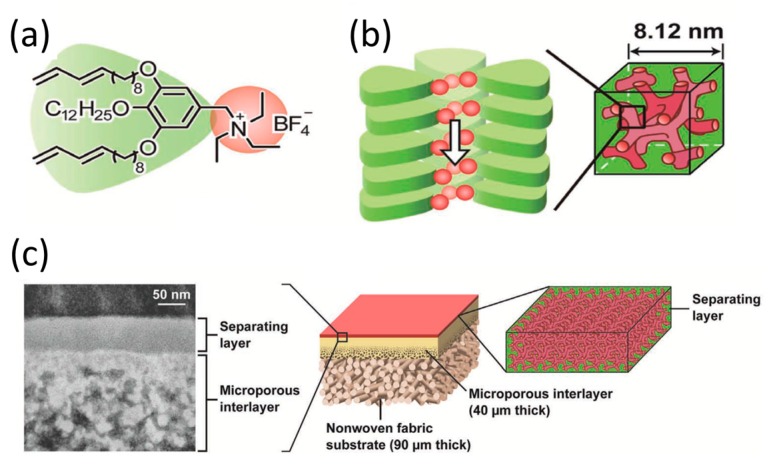

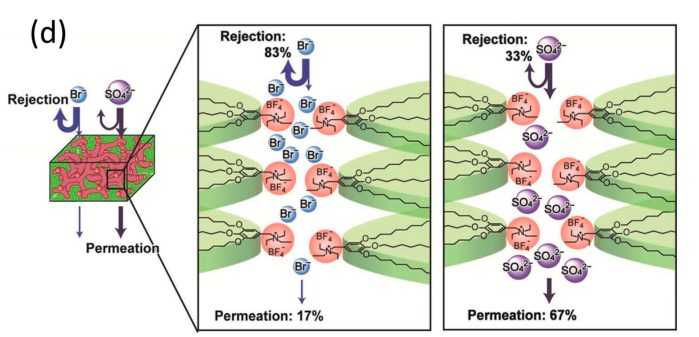
A thin separating layer based on a bicontinuous cubic polymer network. (**a**) Chemical structure of the wedge-shaped liquid crystalline monomer; (**b**) Self-assembled bicontinuous cubic LC phase forming ionic nanochannels; (**c**) Polymer membrane containing the polymerized liquid crystal network as a separating layer; (**d**) Schematic representation of selective rejection of anions through the membrane. Adapted from [89], licensed by John Wiley and Sons, Inc. (Hoboken, NJ, USA).

Similar approaches also found function in breathable barrier materials protecting against chemical agents [87], and in water desalination [88]. Recently, Henmi et al. presented the development of a thin separating layer based on a bicontinuous polymer network with cubic morphology, Figure 13 [89]. The material was constructed from a wedge-shaped amphiphilic monomer with a cationic triethylammonium head. A ~50-nm-thick coating of this material was applied on a microporous support membrane and polymerized to form a selective layer, Figure 13a. This membrane has selective ion-rejection properties; small bromine anions were rejected, while larger sulfate ions could pass through, Figure 13b. In later work, they showed that the material could be used as an active layer on a polysulfone support to reject 20-nm sized viruses from water with high efficiency [90].

## 3. Conclusions and Outlook

This review article highlights recent advances in the field of liquid-crystal-based nanoporous polymers and shows the potential of this type of material for applications. Liquid crystals have successfully been applied to produce nanostructured films, leading to 1D, 2D, and 3D porous polymers. Especially, progress has been made in terms of versatility of the polymerizable LC building blocks, and new methods of curing the different LC phases have been introduced. The LC networks provide a good trade-off between processability and the minimum pore-size that can be achieved. The examples presented in this review demonstrate that new types of nanoporous networks with tailored properties can readily be designed via simple rules. The research field has been brought to a stage where major breakthroughs can be expected. However, several critical challenges must be overcome before applying LC-based nanoporous materials in applications such as adsorbents, ion exchange membranes, filtration, scaffolds for catalytic reactions, conductivity, drug release, and flow batteries. The most notable challenges include control of alignment, pore size, and pore surface chemistry, and the integration of the polymers into functioning devices.

Pore sizes attainable with porous LC polymers have already reached the dimensions required for the nanofiltration of water, and are now even decreasing further towards the level of reversed osmosis membranes. Added benefits of using porous LCs are expected to be increased porosity and monodisperse pores sizes. However, a well-known issue in the fabrication of nanoporous polymers is collapse of the porous structure. The use of LC mixtures with a higher crosslinker content could lead to a more rigid porous material. Altering the shape and size of the LC molecules could also be used to tune the pore size. A totally different approach would be to use plastic crystals (PCs). The increased structural order combined with relatively high molecular mobility makes these crystals excellent building blocks for nanostructured polymer networks. The high degree of order exhibited by PCs will aid applications such as molecular sieving.

Membranes with negatively and positively charged pore walls have been realised, but pore-functionalization is just beginning to be explored. Chemical post-modification is a promising strategy to obtain a wide variety of functionalized pores, and will especially be useful for the selective binding of solutes. In addition to size selectivity and charge selectivity, the introduction of specific non-covalent binding sites in the pores would further increase the specificity with which analytes can be taken up by the porous material. Applications like ion-selective battery separators and membrane design will benefit from a lowering of the capillary pressure and the ability to switch ion-selectivity that pore functionalization may bring about.

Another emerging area of research is the development of responsive or gated nanoporous materials, which can ultimately lead to the active transport of solute. While the incorporation of light-responsive moieties such as azobenzenes has been a fruitful endeavour for inducing colour and shape changes in LC polymers, not much attention has been given to their incorporation in porous LC networks to introduce responsive features such as light-actuated pores [91].

Nanoporous materials based on liquid crystalline polymers have appealing properties that promise to lead to new materials with unprecedented performance. The biggest challenge—the integration of nanoporous LC polymers in devices—still remains. We firmly believe that with the recent advances in LC alignment, realising the true potential of these materials is only a matter of time.
